# Divergent Cross-Adaptation of Herbicide-Treated Wheat and Triticale Affected by Drought or Waterlogging

**DOI:** 10.3390/ijms241512503

**Published:** 2023-08-06

**Authors:** Irina I. Vaseva, Margarita Petrakova, Ana Blagoeva, Dessislava Todorova

**Affiliations:** Institute of Plant Physiology and Genetics–Bulgarian Academy of Sciences, Acad G. Bonchev Str., Bl. 21, 1113 Sofia, Bulgaria; vaseva@bio21.bas.bg (I.I.V.); margarita.p02@abv.bg (M.P.); ani_blagoeva@abv.bg (A.B.)

**Keywords:** antioxidant enzymes, cereal crops, cross-adaptation, ROS, Serrate^®^, water stress

## Abstract

Widely used agrochemicals that do not exert negative effects on crops and selectively target weeds could influence plant resilience under unfavorable conditions. The cross-adaptation of wheat (*Triticum aestivum* L.) and triticale (×*Triticosecale* Wittm.) exposed to two environmental abiotic stressors (drought and waterlogging) was evaluated after treatment with a selective herbicide (Serrate^®^, Syngenta). The ambivalent effects of the herbicide on the two studied crops were particularly distinct in waterlogged plants, showing a significant reduction in wheat growth and better performance of triticale individuals exposed to the same combined treatment. Histochemical staining for the detection of reactive oxygen species (ROS) confirmed that the herbicide treatment increased the accumulation of superoxide anion in the flooded wheat plants, and this effect persisted in the younger leaves of the recovered individuals. Comparative transcript profiling of ROS scavenging enzymes (superoxide dismutase, peroxidase, glutathione reductase, and catalase) in stressed and recovered plants revealed crop-specific variations resulting from the unfavorable water regimes in combination with the herbicide treatment. Short-term dehydration was relatively well tolerated by the hybrid crop triticale and this aligned with the considerable upregulation of genes for L-Proline biosynthesis. Its drought resilience was diminished by herbicide application, as evidenced by increased ROS accumulation after prolonged water deprivation.

## 1. Introduction

The eco-physiological plasticity of economically important crops has gained strategic relevance in light of the ongoing climate crisis. Along with unfavorable environmental conditions, weeds have a deleterious effect on yield, as they are direct competitors for light, nutrients, and water. Due to their higher adaptability, weeds are expected to outcompete crop plants under future climate scenarios characterized by more frequent drought spells, floods, and heat waves [1]. The development of advanced technologies and approaches to counteract the negative consequences of factors that limit staple crops’ productivity become a priority for modern plant breeding [2,3]. The use of complementary strategies to achieve desired traits in crop plants is based on the discovery and subsequent combination of favorable alleles [2]. Bread wheat (*Triticum aestivum*) is grown in diverse environments and represents one of the most important food crops that can ensure global nutritional security [4]. Climate change and intensive agricultural practices have imposed certain challenges for wheat production systems, putting triticale (×*Triticosecale* Wittmack) in the spotlight as an alternative grain crop, especially in regions characterized by limited water availability and climate extremes. Triticale is an intergeneric hybrid of wheat and rye (*Secale cereale*) with improved performance and productivity under less favorable environmental conditions [5]. It combines the high productivity and good grain quality of wheat with greater abiotic stress tolerance and undemanding soil fertility requirements of rye. Variations in the stress tolerance of different triticale cultivars have also been documented, especially in their ability to withstand low temperatures [5].

Wheat and triticale initiate the tillering phase of their development during winter, and weed control in early vegetation has a significant effect on the subsequent productivity of these economically important crops [6]. In this aspect, the application of post-emergence herbicides is a common agricultural practice in cereal crops. Triticale can be successfully treated with agrochemicals designed for use on wheat [7]. Integrated pest management measures, like crop rotations or the use of biological agents, which are capable of reducing the input of chemical compounds in food production, will be the preferable approach in the future [8,9], but synthetically produced herbicides continue to be the mainstay in traditional cropping systems [10]. The herbicide Serrate^®^ (Syngenta) is a wide-spectrum herbicide and is highly efficient for the control of compact brome, a weed that impacts cereal crops causing huge yield losses. The product has been registered for application on wheat, rye, and triticale and comprises two active components—clodinafop-propargyl and pyroxsulam. Clodinafop-propargyl inhibits the activity of acetyl co-enzyme A carboxylase, associated with fatty acid biosynthesis. Its herbicide action against annual grass weeds is well established. One of its advantages is that it tends not to be environmentally persistent and is not expected to leach into groundwater. Pyroxsulam is an acetolactate synthase inhibitor. The two active compounds of Serrate^®^ are routinely used in wheat crop cultivation [11,12,13].

Herbicides are regarded as a significant factor of abiotic stress, as their action interferes with proteins, enzymes, and other essential elements of primary and secondary metabolism, causing oxidative stress in plants [14,15,16]. The buildup of harmful ROS, like superoxide radical (O_2_^●−^) and hydrogen peroxide (H_2_O_2_), in plant tissues experiencing physiological stress is prevented by the action of enzymes from the antioxidative defense system, represented by superoxide dismutase (SOD, EC 1.15.1.11), catalase (EC 1.11.1.6), and peroxidase (EC 1.11.1.7) [12]. SOD enzymes partition O_2_^●−^ into O_2_ and H_2_O_2_, whereas catalase and peroxidase are responsible for reducing cellular H_2_O_2_ levels. Glutathione reductase (GR, EC 1.6.4.2) plays an essential role in the defense system against ROS by regenerating the glutathione pool using NADPH as the electron donor [17,18]. GR is active in chloroplasts, cytoplasm, peroxisomes, and mitochondria. CAT enzymes, which also handle excessive amounts of hydrogen peroxide in the cell, are found only in peroxisomes, whereas POX enzymes operate in the cell walls and cytoplasm as well, where they oxidize aromatic electron donors like guaiacol and pyrogallol at the expense of H_2_O_2_.

Herbicide application upregulates genes associated with antioxidant defense and stress acclimation [19]. Consequently, the nature of the herbicide priming effect has mainly been associated with the alteration of the regulations of stress-inducible genes [14,15,20,21]. Previous studies have also demonstrated that drought activates the expression of particular *SODs* [22] and catalase-coding genes [23], as well as the accumulation of transcripts associated with the biosynthetic pathway of proline [24,25]. Information on the comparative antioxidant gene expression in wheat and triticale subjected to combined stress provoked by herbicide application and water stress is limited. Therefore, the aim of the present study was to evaluate the occurrence of cross-adaptation and the cross-synergistic response of herbicide-preconditioned winter wheat and triticale to subsequent drought and flooding. We estimated the accumulation of ROS and the expression of genes coding antioxidant enzymes in the leaf tissue of the stress-affected and recovered individuals. The transcript profiles of genes coding for SODs [22,26], catalases [23], peroxidases [27,28], glutathione reductase [29], and proline-related enzymes [24,25] were also monitored.

## 2. Results

Both crops were grown in soil under controlled conditions. The standard herbicide dose was applied to part of the plants when the second true leaf was fully expanded. Seventy-two hours later, the stress program was initiated. It comprised flooding or water deprivation of plants that did or did not receive Serrate^®^ treatment. The data were collected at four sampling points: 72 h after the treatment with herbicide; 96 h after the initiation of the stress program; 168 h of stress; and 96 h after resuming the normal water regime (recovery stage). The phenotypes of the different experimental groups are represented in Figure S1, and the experimental model is depicted in Figure S2.

### 2.1. Effect of Drought and Waterlogging on Growth of Wheat and Triticale Pretreated with Selective Herbicide

**Figure 1:**
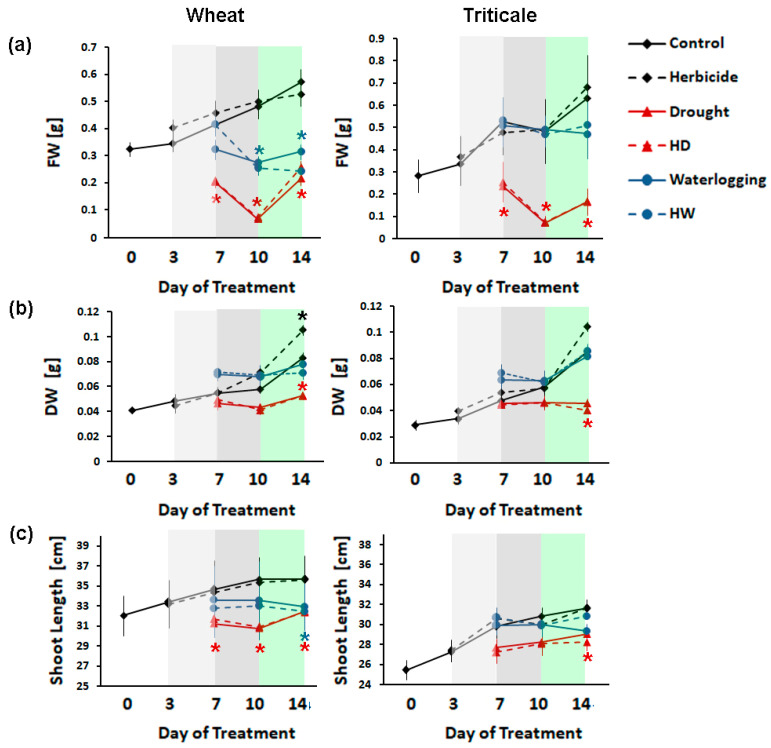
Growth parameters: (**a**) fresh weight (FW), (**b**) dry weight (DW), and (**c**) shoot length of control (solid black line), herbicide-treated (dashed black line), drought-stressed (solid red line), waterlogged plants (solid blue line), and plants subjected to combined stress treatments (HD—herbicide and drought, designated by dashed red line; HW—herbicide and waterlogging, designated by dashed blue line). Results from four sampling points are represented: 72 h after herbicide application (day 3, white area on the graphs); 96 h of stress (day 7, light-gray area), 168 h of stress (day 10, dark-gray area), and after 96 h of recovery (day 14, light-green area). The bars represent the standard deviation (SD, *n* ≥ 15). Asterisks designate statistically significant differences among the treatment groups at each sampling point (at *p* < 0.05; one-way ANOVA with Tukey HSD analyses that are shown in Tables S1 and S2).

The measurements of the aboveground length and fresh and dry weights outlined some distinct features in the stress responses of the two tested crops and how they were affected by the applied herbicide (Figure 1).

Overall, the fresh weight (FW) of the aboveground plant parts was not influenced by the standard herbicide treatment (Figure 1a). The DW difference was detected at the recovery sampling point in the herbicide-sprayed triticale experimental group, which was grown under normal conditions, but it was not significantly different after Tukey’s HSD test. The dynamic changes in the fresh weight showed that there were differences between the wheat and triticale individuals (Figure 1a). Drought-stressed triticale and wheat plants maintained a similar FW in both groups (herbicide-treated and non-treated plants). It was unexpected to observe that drought-stressed wheat plants recovered their fresh weight slightly better than the respective triticale group after resuming the normal water regime. More distinct differences between the two crops were documented in the FW of individuals subjected to waterlogging, with triticale showing an increase in this parameter upon recovery (Figure 1a). The pre-stress herbicide application had no additional effect on the FW changes of the flooded wheat and triticale plants (Figure 1a).

The ability of herbicides to stimulate plant growth has been previously documented [30,31]. We observed a similar effect on wheat and triticale plants that have received the recommended Serrate^®^ dose and were grown under optimal conditions. The dry weight (DW) of the herbicide-treated individuals (H) of the wheat experimental group showed a statistically significant increase of 20% at the end of the experimental period (Figure 1b).

The stronger negative consequences of waterlogging on the accumulation of biomass in herbicide-treated wheat were demonstrated by the reduced FW after prolonged exposure to excessive water (Figure 1a). This crop was more severely affected by the waterlogging, judging from the lack of FW change at the recovery stage. The observed negative effect was comparable in the plants that received the standard Serrate^®^ dose. Triticale plants that were subjected to waterlogging exhibited a relatively smaller drop in FW compared to the same treatment group of *T. aestivum* (Figure 1a).

The length of the aboveground parts of wheat plants subjected to both types of stress remained lower than the controls (Figure 1c). Similarly, the drought-affected triticale individuals showed reduced elongation growth, but this was less pronounced and statistically significant for the plants subjected only to the combination of drought and Serrate^®^ application (HD) at the recovery sampling point (Figure 1c).

In summary, the monitored growth parameters of the two crops confirmed the relatively better tolerance of triticale toward unfavorable water supply (both excessive and limited) but also outlined that the application of a selective herbicide might negatively affect its performance under prolonged drought stress.

### 2.2. Accumulation of Reactive Oxygene Species (ROS)

#### 2.2.1. ROS Detection in Wheat Subjected to Drought or Waterlogging after Herbicide Treatment

**Figure 2:**
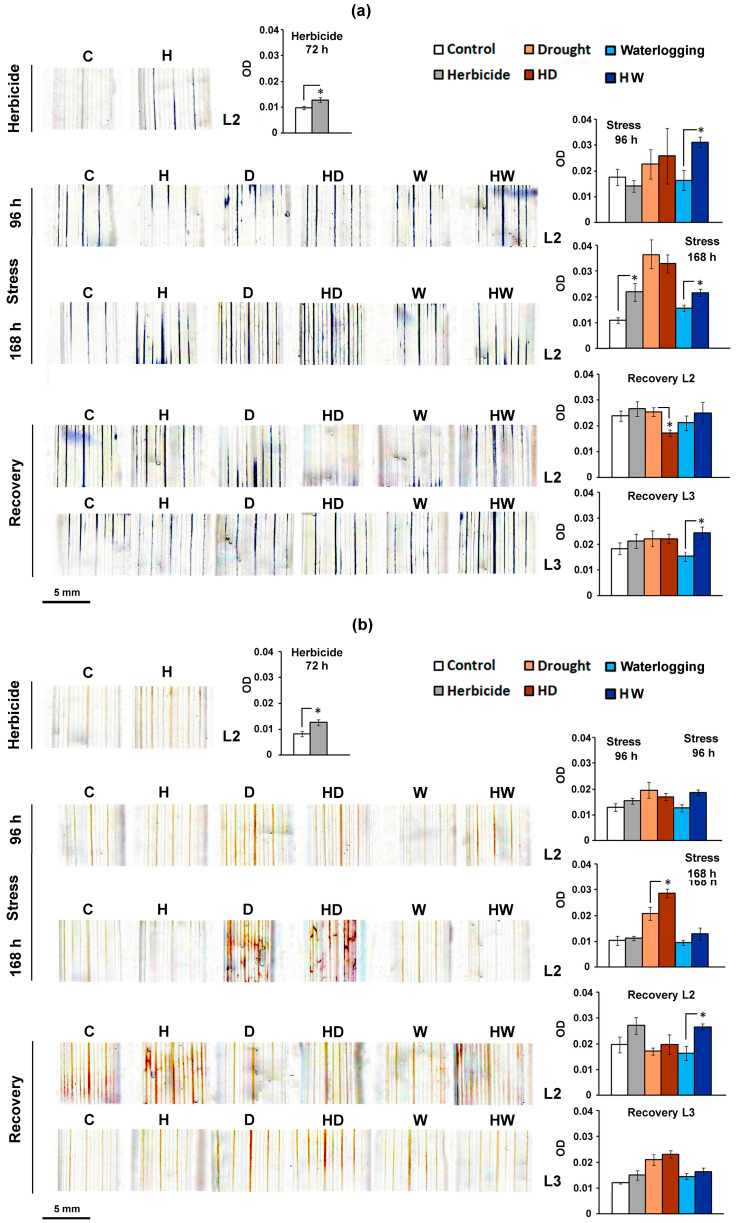
Histochemical staining for the detection of ROS in the last fully expanded true leaf of wheat control plants (C) and plants subjected to a standard dose of a selective herbicide (H), drought (D) or waterlogging (W), and their combinations (HD and HW, respectively). The second leaf (L2) was used at each sampling point, and the third leaf (L3) was included at the recovery stage. (**a**) NBT staining was applied for the detection of superoxide anion (O_2_^●−^), and (**b**) hydrogen peroxide (H_2_O_2_) accumulation was detected via DAB staining. The graphs represent the optical density (OD) with the calculated standard errors (SE, *n* ≥ 3). The asterisks (*) designate statistical significance between the compared experimental groups (*p* < 0.05, one-way ANOVA).

The histochemical staining for the detection of ROS showed that wheat plants tended to accumulate transiently higher O_2_^●−^ (Figure 2a) and H_2_O_2_ amounts (Figure 2b) in the second fully expanded true leaf (L2) after being treated with the standard Serrate^®^ dose.

##### Drought-Affected Wheat

As expected, the accumulation of harmful O_2_^●−^ in the leaves of drought-stressed plants accelerated with the progress of water limitation (samples designated by the letter D). The herbicide pretreatment did not significantly change the superoxide staining intensity of the drought-stressed individuals (HD samples) (Figure 2a). After the period of recovery, the low NBT-staining intensity of L2 derived from HD wheat plants was mainly due to the dramatically decreased leaf viability (Figure 2a).

The histochemical staining for the detection of hydrogen peroxide showed that the leaves of dehydrated plants accumulated higher amounts of H_2_O_2_ after prolonged stress (at 168 h), and the herbicide treatment significantly enhanced the DAB staining of HD wheat samples (Figure 2b). After normalizing the water supply, the hydrogen peroxide content in D and HD samples derived from L2 dropped to the control levels but remained higher in the younger L3 leaves, reflecting the effect of the experienced stress.

##### Waterlogged Wheat

The NBT-staining intensity of the samples that experienced only excessive water stress (W) was comparable to that of the control (Figure 2a). However, the waterlogged plants that received herbicide treatment (HW) were more prone to the accumulation of higher amounts of harmful O_2_^●−^ in the leaves.

Both the W and HW experimental groups did not exhibit any significant changes in the hydrogen peroxide content during stress, but HW individuals exhibited considerable DAB staining of L2 samples upon recovery (Figure 2b). The younger leaves that reached full expansion after recovery (L3) in the HW group had higher O_2_^●−^, which was visualized upon NBT staining.

#### 2.2.2. ROS Detection in Triticale Subjected to Drought or Waterlogging after Herbicide Treatment

**Figure 3:**
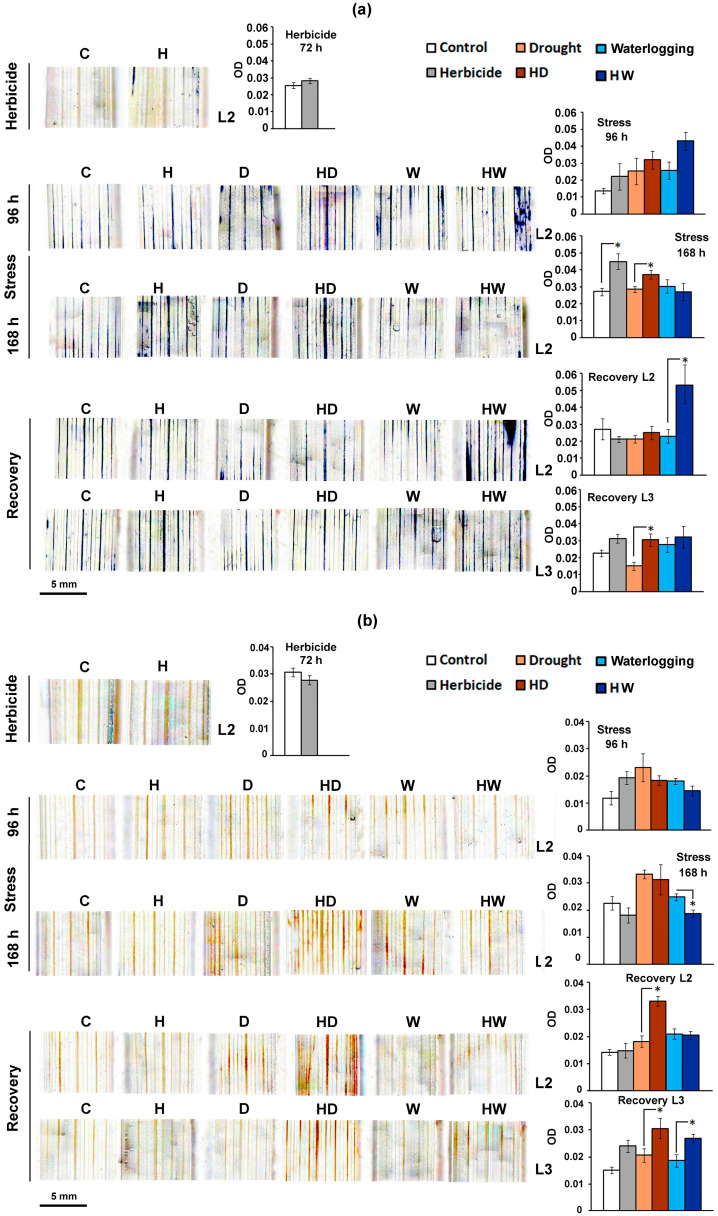
Histochemical staining for the detection of ROS in the last fully expanded true leaf of triticale control plants (C) and plants subjected to a standard dose of a selective herbicide (H), drought (D) or waterlogging (W), and their combinations (HD and HW, respectively). The second leaf (L2) was used at each sampling point, and the third leaf (L3) was included at the recovery stage. (**a**) NBT staining was applied for the detection of superoxide anion (O_2_^●−^), and (**b**) hydrogen peroxide (H_2_O_2_) accumulation was detected via DAB staining. The graphs represent the optical density (OD) with the calculated standard errors (SE, *n* ≥ 3). The asterisks (*) designate statistical significance between the compared experimental groups (*p* < 0.05, one-way ANOVA).

In contrast to the observations made in wheat, the standard Serrate^®^ dose did not induce ROS accumulation in the leaves of triticale (L2) shortly after the treatment (H samples at 72 h) (Figure 3a,b). This was indicative of a possible divergent effect of the selective herbicide on the two crops upon subsequent stress exposure. A delayed, gradual increase in the superoxide anion levels in the H samples was observed, reaching statistically significant values at the sampling point corresponding to 168 h of stress (Figure 3a).

##### Drought-Affected Triticale

Despite its previously acknowledged drought tolerance [32], prolonged water limitation (168 h of stress) considerably affected triticale growth (Figure 1), but the histochemical ROS detection showed some distinct crop-specific patterns (Figure 3a,b). A rise in O_2_^●−^ level was detected in the HD triticale experimental group, but not in the “drought-stressed-only” individuals (D) (Figure 3a). This suggests that the observed effect likely results from the herbicide application before the stress period, rather than dehydration. Increased H_2_O_2_ accumulation in the leaves of drought-affected triticale was also detected after prolonged stress (at 168 h), and the DAB-staining intensity of the D and HD samples was similar, suggesting that the herbicide treatment did not influence this parameter (Figure 3b).

Later, at the recovery stage, H_2_O_2_ levels of the D samples had already dropped to those of the controls but apparently, the herbicide treatment provoked the maintenance of higher amounts of hydrogen peroxide in triticale leaves, even after rehydration, as evident in the DAB-staining patterns of both L2 and L3 of the HD samples (Figure 3b). The water-limiting conditions seemed to provoke a slightly higher superoxide anion content in the younger triticale leaves (L3) of the HD experimental group (Figure 3a), but the levels were comparable to those of the control plants that received the standard herbicide treatment (H).

##### Waterlogged Triticale

The waterlogged triticale plants (W) did not exhibit elevated levels of superoxide anion (Figure 3a) or hydrogen peroxide (Figure 3b), which corresponded to their better physiological status compared to the dehydrated triticale and the flooded wheat plants (Figure 1).

A transient higher O_2_^●−^level was detected at 96 h of stress in the HW experimental group. The prolonged flooding of the HW triticale plants likely invoked an adaptive response, since the accumulation of the superoxide anion in this experimental group dropped to that of the control level (C) at 168 h of stress. In line with this assumption was the observed drop in H_2_O_2_ levels in the HW compared to the W triticale plants, visualized by DAB staining (Figure 3b).

After recovery, the intensive NBT staining of L2 of the HW triticale plants corresponded to accelerated senescence, which is usually accompanied by the accumulation of ROS, including superoxide anion. On the other hand, the detected O_2_^●−^ content in the younger L3 in the recovered-from-stress HW individuals was similar to that of the controls. A reciprocal observation was made for H_2_O_2_ content of the recovered HW triticale plants—the DAB staining had slightly higher intensity in the younger leaves (L3), but the level was comparable to that detected in the respective herbicide control (H).

### 2.3. Transcript Profiling of Genes Coding for ROS Scavenging Enzymes in the Leaves of Wheat and Triticale Subjected to Drought and Waterlogging after the Application of a Selective Herbicide

In green plant tissues, chloroplasts are considered a major site for the production of ROS because molecular oxygen can serve as an electron acceptor at the reducing site of photosystem I (PS I). ROS accumulation in the leaves of wheat and triticale subjected to herbicide treatment and subsequently exposed to unfavorable water supplies showed some distinct features, which anticipate a crop-specific activation of genes coding for components of the antioxidative cellular defense. We profiled the transcript accumulation of several *T. aestivum* antioxidant enzyme-coding genes in the leaves of wheat and triticale in search of differential activation of their expression in the genomes of the two crops.

#### 2.3.1. Expression of SOD-Coding Genes in Wheat and Triticale Subjected to Drought or Waterlogging in Combination with Herbicide Treatment

**Figure 4:**
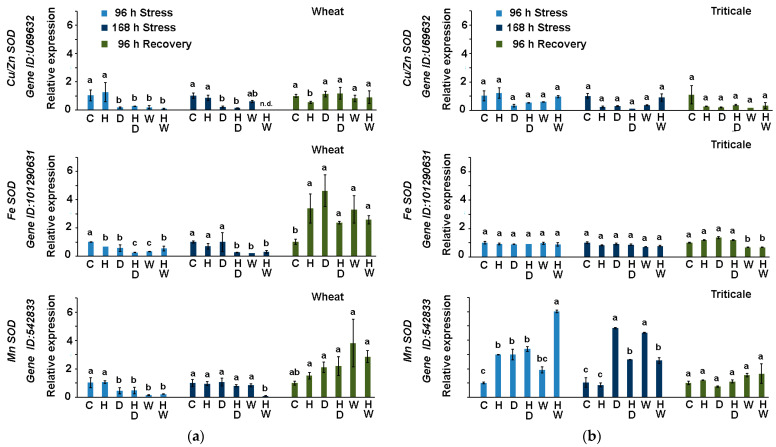
Transcript profiling of genes coding for *T. aestivum Cu/ZnSOD*, *FeSOD*, and *MnSOD* genes in wheat (**a**) and triticale (**b**) leaves derived from control (C), herbicide-treated (H), drought-stressed (D), waterlogged plants (W), and plants subjected to combined stress treatments (HD—herbicide and drought; HW—herbicide and waterlogging) at different sampling points (96 h of stress, 168 h of stress, after 96 h of recovery). Values are means of three biological repeats (*n* = 3) ± SD; n.d.—“not detected”. The lowercase letters designate statistically significant differences among the treatments at each sampling point (*p* < 0.05, one-way ANOVA).

There are several SOD isoforms with specific subcellular localization [33]. Mn-SODs function in the mitochondrial matrix and peroxisomes, and Cu/Zn-SODs are active in the chloroplast, cytosol, and extracellular space. Fe-SOD isoforms operate mostly in the chloroplast. Previously, it has been observed that *T aestivum MnSOD* genes are drought inducible and usually decrease after rehydration [22]. In the present study, comparative transcript profiling outlined *T. aestivum MnSOD* gene divergent expression between wheat and the hybrid crop triticale (Figure 4).

We detected a higher relative transcript abundance of the gene in triticale leaves at the two sampling points of the stress treatment (Figure 4b). In comparison, this *MnSOD* gene was moderately activated, mainly in the recovered-from-stress wheat plants (Figure 4a). The herbicide application was found to positively affect the level of *MnSOD* expression under short-term waterlogging of triticale plants (Figure 4b). Later on, at 168 h of stress, the expression of *MnSOD* showed a sustained increase in triticale D and W samples. *MnSOD* transcript abundance in the HD and HW experimental groups was lower compared to the stressed plants that were not treated with herbicide, but the expression levels remained approximately twofold higher than in the controls. The observed induced expression of *MnSOD* under unfavorable conditions in triticale leaves could be linked to its improved ability to detoxify the free radical superoxide, which is a major by-product of mitochondrial respiration.

The expression of *T. aestivum FeSOD* has relatively low expression levels and appears to be stable under different environmental abiotic stresses [26]. In our study, the hybrid crop triticale (Figure 4b) also exhibited a stable accumulation of *FeSOD* transcript in all experimental groups at each sampling point (96 h and 168 h of drought or waterlogging stress, as well as after recovery). After recovery, the levels of expression of this particular transcript were approximately threefold higher in wheat samples compared to the levels detected in triticale (Figure 4a,b). During the stress period, an inhibition of *FeSOD* transcript accumulation was detected in wheat HD, W, and HW samples (Figure 4a), which was not observed in the respective triticale samples (Figure 4b).

In a previous study, it was observed that drought did not provoke a higher expression of *Cu/ZnSOD,* but the gene was upregulated after rehydration [22]. The expression levels of this gene were also relatively less affected in the present study. The differences in *Cu/ZnSOD* transcript profiles between wheat and triticale samples were not as distinct as those identified for the other two SOD genes. However, it was noted that in triticale HW samples, its expression was similar to that of the control, even after 96 h and 168 h of stress. In contrast, the HW wheat samples were characterized by strongly inhibited *Cu/ZnSOD* levels.

#### 2.3.2. Expression of *T. aestivum* Genes Coding for Catalase and Peroxidase in Wheat and Triticale Subjected to Drought or Waterlogging in Combination with Herbicide Treatment

**Figure 5:**
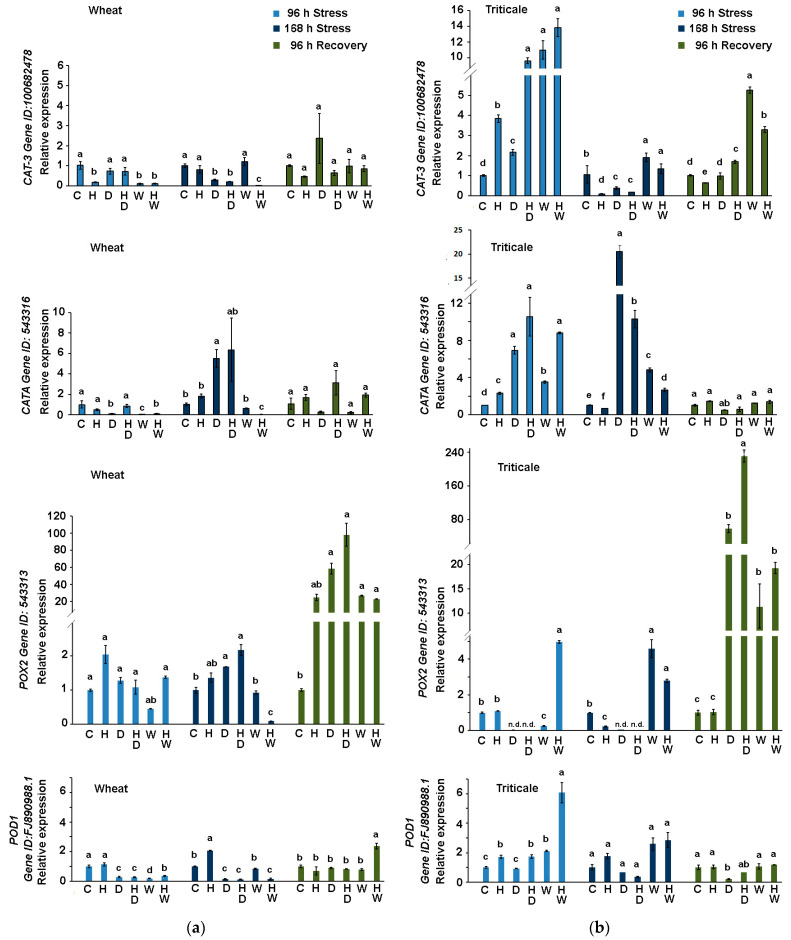
Transcript profiling of *T. aestivum* catalase (*CAT-3* and *CATA*) and peroxidise genes (*POX2* and *POD1*) in wheat (**a**) and triticale (**b**) leaves derived from control (C), herbicide-treated (H), drought-stressed (D), waterlogged plants (W), and plants subjected to combined stress treatments (HD—herbicide and drought; HW—herbicide and waterlogging) at different sampling points (96 h of stress, 168 h of stress, after 96 h of recovery). Values are means of three biological repeats (*n* = 3) ± SD; n.d.—“not detected”. The lowercase letters designate statistically significant differences among the treatments at each sampling point (*p* < 0.05, one-way ANOVA).

Hydrogen peroxide is a reactive oxygen species with a recognized signaling role [34,35]. Its physiologically relevant levels are maintained by the enzymes catalase and peroxidase, which detoxify the excessive H_2_O_2_ amounts. We monitored the changes in the expression of two catalase and two peroxidase coding genes in the leaves of wheat (Figure 5a) and triticale (Figure 5b) subjected to unfavorable water regimes, assessing the additional effect of a standard herbicide dose applied prior to stress.

The abundance of catalase transcripts (*CAT-3* and *CATA*) in the leaves of the triticale plants subjected to drought and waterlogging was considerably higher (Figure 5b) than that measured in wheat (Figure 5a), suggesting elevated synthesis of the enzyme for the timely detoxification of excessive amounts of hydrogen peroxide. Catalase gene expression was stimulated in the control triticale plants that received a standard herbicide dose (H), with a particularly distinct upregulation of *CAT-3* transcripts (Figure 5b). The application of the herbicide seemed to promote even higher expression for both monitored catalase genes (Figure 5b) during the early stress phase (at 96 h of stress). Relatively higher *CAT-3* expression was also detected in triticale individuals recovering from excessive water stress, with lower levels measured in the HW samples compared to the W experimental group.

We followed the expression of *T. aestivum* genes coding for ascorbate (*POD1*) and guaiacol (*POX2*) peroxidases in wheat (Figure 5a) and triticale (Figure 5b). Overall, at 96 h of stress *POD1* transcript levels were maintained slightly higher (HD and W) or close to the controls (D), with a considerable rise observed in triticale HW experimental group (Figure 5b).

A sustained higher abundance of *POD1* and *POX2* transcripts was also measured in triticale plants at 168 h of waterlogging (Figure 5b), which coincides with the relatively better performance of the hybrid under this type of stress compared to wheat experimental group that received the same treatment. During stress, *POX2* transcripts exhibited relatively stable levels in wheat samples derived from the dehydrated individuals (D and HD) (Figure 5a), whereas they were undetectable in the respective triticale samples (Figure 5b). A very strong induction of *POX2* expression after recovery was registered in the samples derived from both crops, with levels exceeding 60-fold (Figure 5a) and 80-fold (Figure 5b) in the drought-stressed (D) wheat and triticale, respectively. The herbicide treatment appeared to be a synergistic element regarding the high accumulation of *POX2* transcripts after rehydration, which was a consistent observation in both HD groups of wheat and triticale (Figure 5a,b). High *POX2* transcript abundance was measured in wheat and triticale plants recovering from excessive water stress (W and HW experimental groups), but the estimated levels did not exceed 20-fold in both crops.

#### 2.3.3. Expression of *T. aestivum* Glutathione Reductase Gene in Wheat and Triticale Subjected to Drought or Waterlogging in Combination with Herbicide Treatment

**Figure 6:**
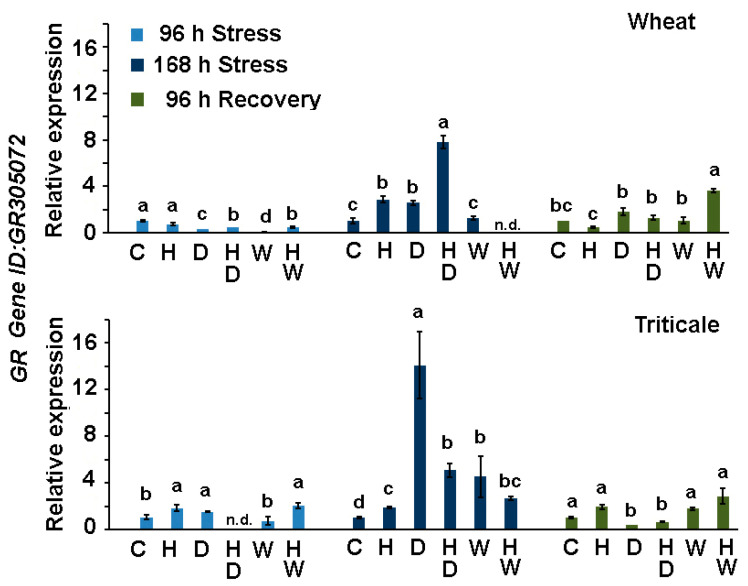
Transcript profiling of *T. aestivum* glutathione reductase gene (*GR*) in wheat and triticale leaves derived from control (C), herbicide-treated (H), drought-stressed (D), waterlogged plants (W), and plants subjected to combined stress treatments (HD—herbicide and drought; HW—herbicide and waterlogging) at different sampling points (96 h of stress, 168 h of stress, after 96 h of recovery). Values are means of three biological repeats (*n* = 3) ± SD; n.d.—“not detected”. The lowercase letters designate statistically significant differences among the treatments at each sampling point (*p* < 0.05, one-way ANOVA).

Glutathione reductase (GR) maintains a supply of the reduced form of glutathione, which is the most prevalent cellular antioxidant participating in the detoxification of excessive amounts of H_2_O_2_ generated during stress. Comparative expression analyses of a *T. aestivum GR* gene (GenBank: GR305072) in the leaves showed that triticale accumulates considerably higher transcript amounts compared to wheat under drought stress (Figure 6). The pre-stress herbicide treatment had divergent effects on *GR* expression levels in the dehydrated wheat and triticale plants, which became apparent at 168 h of drought. Wheat plants that received a standard Serrate^®^ dose and were subjected to water limitation (HD) had higher *GR* transcript levels compared to the group of drought-stressed plants that were not treated with the herbicide (D). An opposite herbicide effect on *GR* expression was observed in triticale HD samples.

The transcript abundance in the waterlogged triticale (W) exhibited a moderate increase and remained relatively stable in the HW-treated plants, unlike the trend observed in the same experimental group of wheat, where GR expression was strongly inhibited. This observation coincides with the more pronounced wheat vulnerability under combined HW stress compared to triticale, which better handled the combination of herbicide treatment with subsequent excessive water stress.

#### 2.3.4. Expression of *T. aestivum* Genes for Enzymes from the L-Proline Biosynthesis in Wheat and Triticale Subjected to Drought or Waterlogging in Combination with Herbicide Treatment

Under dehydration, plant cells usually accumulate free proline (L-Pro) as an osmoprotectant. Regardless of its relatively modest antioxidant activity, the presence of L-Pro in high amounts may contribute to the ROS scavenging network operating inside the cell, as it is capable of quenching singlet oxygen and superoxide anion [36].

**Figure 7:**
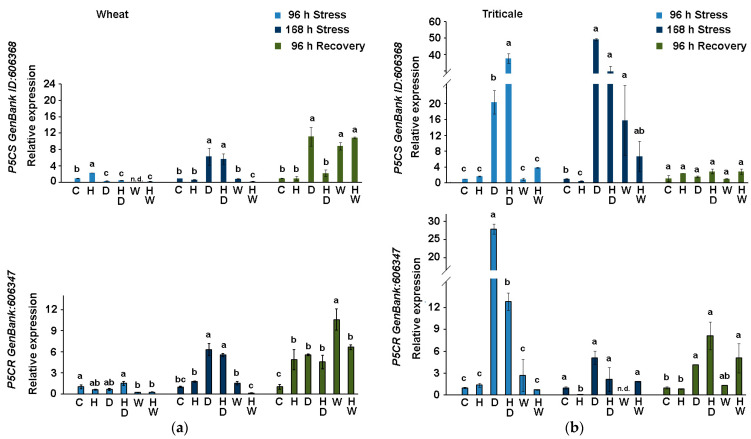
Transcript profiling of *T. aestivum* delta-1-pyrroline-5-carboxylate synthase (*P5CS*) and pyrroline-5-carboxylate reductase (*P5CR*) genes in wheat (**a**) and triticale (**b**) leaves derived from control (C), herbicide-treated (H), drought-stressed (D), waterlogged plants (W), and plants subjected to combined stress treatments (HD—herbicide and drought; HW—herbicide and waterlogging) at different sampling points (96 h of stress, 168 h of stress, after 96 h of recovery). Values are means of three biological repeats (*n* = 3) ± SD; n.d.—“not detected”. The lowercase letters designate statistically significant differences among the treatments at each sampling point (*p* < 0.05, one-way ANOVA).

We monitored the expression of two genes from proline biosynthesis: delta-1-pyrroline-5-carboxylate synthase (*P5CS*) and pyrroline-5-carboxylate reductase (*P5CR*). P5CS catalyzes the limiting step of L-Pro biosynthesis. The transcript profiling of these two genes showed that the drought-stressed triticale had considerably higher *P5CS* and *P5CR* transcript contents (Figure 7b) compared to those measured in the similarly treated wheat plants at 96 h of stress (D and HD) (Figure 7a). Persistent upregulated gene expression in triticale samples was also registered at 168 h of drought stress, with particularly higher *P5CS* levels compared to the same experimental groups of wheat. It should also be noted that *P5CS* gene was upregulated in the leaves of the waterlogged triticale, unlike the low levels of its expression registered in the same treatment group of wheat plants (W and HW).

Treatment with a standard dose of herbicide did not significantly affect the levels of *P5CS* and *P5CR* expression in the drought-stressed wheat (Figure 7a), whereas at 168 h of stress, reduced transcript levels of both genes were registered in the HD triticale samples (Figure 7b).

During the recovery period, *P5CR* remained upregulated in most wheat (Figure 7a) and triticale samples (Figure 7b). The only exception was observed for the recovered-from-stress W triticale group, where the level of *P5CR* expression was comparable to that of the control samples. Similarly, *P5CS* transcript levels in all triticale samples recovering from drought (D, HD) or waterlogging (W, HW) were closer to those of the control (Figure 7b). In contrast, the recovered-from-stress wheat plants still exhibited high *P5CS* expression, except in the HD experimental group, which showed diminished transcript content (Figure 7a).

## 3. Discussion

The induction of improved plant tolerance to future stress exposure has been suggested as a possible outcome of herbicide application [37,38]. The potential of routinely used herbicides to invoke complementary stress priming in cereal crops has been proposed based on the observed activation of the different components of antioxidative defense [20,38,39] and acclimation [19] after the treatment. The design of the experimental model in our study addresses the combined effects of adverse water regimes and herbicide application at the early vegetative stage of winter wheat and triticale. The data obtained in the present study add some new details regarding the herbicide preconditioning effects, for which existing knowledge is still rather limited [30]. Previously, it has been suggested that herbicides could cause reversible short-term enhancement of plant vegetative fitness [30]. We observed a similar effect resulting from the application of the recommended Serrate^®^ dose on both wheat and triticale. The individuals that received the herbicide and were grown under optimal conditions had higher dry-mass accumulation at the end of the experimental period compared to the control plants. However, we also registered negative consequences occurring after the application of a standard herbicide dose, followed by exposure to an adverse water supply.

### 3.1. Herbicide Treatment Reduces Triticale Tolerance to Prolonged Drought and Enhances the Negative Effect of Waterlogging on Wheat

It has been already established that herbicide action could be modified under unfavorable environmental conditions, like varying temperatures [40], drought [41], and high salinity [42]. Weed control in waterlogged fields resulting from heavy precipitation at the beginning of vegetation is particularly unpredictable. Crop seedlings growing under cold/wet conditions at this developmental stage may be more sensitive to herbicide injury due to the combination of hypoxia and suboptimal temperatures. The present study outlines the negative cross-synergism of herbicide application followed by waterlogging on wheat, and it also demonstrates that triticale is better suited for withstanding such combined stress. Meanwhile, we confirmed the reduced tolerance of triticale toward prolonged drought if a standard herbicide dose has been previously applied. Indeed, triticale cultivars are characterized by large genetic diversity, ensuring increased physiological plasticity and adaptability potential. However, the results from this study once again point out that the abiotic stress resistance of this relatively resilient grain crop cannot be taken a priori without proper elucidation [5].

### 3.2. The Expression of Wheat Antioxidant Genes Shows Divergent Profiles in Hybrid Crop Triticale Subjected to Stress

Most of the presently grown bread wheat varieties are alloploid and contain several subgenomes [43]. This implies that the coordination of homoeologous gene expression at the co-transcriptional level is rather complex [43,44]. It has already been demonstrated that polyploidy affects the gene expression patterns in bread wheat, and these are linked to epigenetic changes and variations in transposable elements within the promoters of homoeologous genes [43]. The gene expression regulatory network is expected to be even more complex in the hybrid landscape of *Triticosecale,* implementing unique features that could trigger divergent physiological and stress-adaptive responses.

The distinct stress preconditioning properties of the standard herbicide dose on the two crops became evident shortly after the application of the weed management product. We observed upregulation of the catalase gene (*CAT-3*) and the SOD-coding gene (*Mn-SOD*) in triticale H samples 72 h after spraying, and this coincided with the relatively stable ROS levels identified there. This was in contrast to the respective wheat experimental group, where substantial changes in the expression of the monitored genes were not registered, but a rise in ROS accumulation did occur.

The distinct expression patterns of genes coding for antioxidant enzymes in wheat and triticale became more obvious as the stress advanced. The stable expression of glutathione reductase, catalase, and peroxidase genes in the waterlogged triticale contrasted with the reduced transcript abundance of the same genes measured in wheat W and HW samples. This observation could be associated with the better performance of the hybrid crop triticale under excessive water stress. Moreover, wheat plants subjected to combined HW treatment had lower dry weights compared to those solely experiencing waterlogging. Our previous work has demonstrated that Serrate^®^ has an insignificant effect on photosynthesis-related parameters in wheat [45]. Therefore, the negative cross-synergism of the herbicide and flooding, manifested by reduced wheat dry weight, could be attributed to enhanced oxidative stress occurring in the HW experimental group.

### 3.3. Herbicide-Treated Wheat and Triticale Recovery Patterns in Individuals Affected by Drought and Waterlogging

The data obtained from the post-stress phase demonstrate the plant’s capacity to recover and resume normal growth after experiencing adverse environmental conditions, and therefore, these have high informative value [46]. The vulnerability of wheat toward excessive water stress in combination with herbicide application was manifested by the persistently elevated ROS content in the leaves of recovered plants. This corresponds to the observed higher stress-marker levels in this experimental group [47]. ROS histochemical staining of the HW-treated wheat showed that the younger leaves (L3) developed after the stress period have high superoxide anion levels, an effect that was not registered in the W experimental group. The inhibition of the expression of the monitored SOD genes in these individuals corresponded with the reduced capacity of the plants to handle the high amounts of superoxide anion. In comparison, the respective triticale HW plants exhibited relatively stable superoxide anion-staining patterns, along with unaffected expression of SOD genes (*Cu/ZnSOD* and *FeSOD* in particular). The transcript abundance of *MnSOD* in the same treatment group even increased at the beginning of the stress period.

Meanwhile, the increased vulnerability of herbicide-treated triticale toward prolonged water deprivation (HD sample group at 168 h of drought stress) was correlated with the reduced expression of some of the monitored genes, particularly the genes from the biosynthetic pathway of proline-, catalase-, and Mn-SOD-coding ones. The negative effect of the combined drought/herbicide treatment on triticale was also manifested by the measured lower fresh and dry weights upon recovery. This was accompanied by higher H_2_O_2_ levels in both L2 and L3 of the recovered-from-stress HD triticale individuals, suggesting ongoing oxidative distress in these leaves.

Usually, the older leaves, as well as those damaged by the experienced physiological stress, are the ones that senesce quickly upon recovery, which is one of the means to remobilize resources [48]. At the end of the experimental period, the older leaves (L2) in wheat control plants (both C and H), unlike triticale individuals, showed signs of natural senescence, manifested by the higher accumulation of ROS, as evident in the NBT- and DAB-staining patterns. Upon recovery from waterlogging, the accelerated senescence in the older (L2) HF leaves coincided with elevated hydrogen peroxide in wheat and O_2_^●−^ in triticale. An interesting reciprocal ROS “stress memory mark” in the younger leaves (L3) was identified: the last fully expanded leaves had higher H_2_O_2_ levels in triticale and higher O_2_^●−^levels in wheat HW group. It is known that the ratio of O_2_^●−^ and H_2_O_2_ is dependent on the types of substrates that are oxidized to drive the electron transport chain [49]. Therefore, it may be assumed that the composition of substrates related to electron transport in senescing and recovered leaves of HW triticale and wheat differs. Such a possibility requires additional investigation, as it may provide important details on the crop-specific characteristics of the electron transport chain function of triticale under stress, explaining its better capacity to sustain energy levels and detoxify the generated reactive oxygen species.

## 4. Materials and Methods

### 4.1. Plant Material and Treatments

The seeds of *Triticum aestivum* L., cv. Sadovo-1 (a standard winter wheat cultivar) and *Triticosecale* Wittm., cv. Rojen (a rye-type triticale cultivar), were provided by the Institute of Plant Genetic Resources (Sadovo, Bulgaria). In previously performed field experiments, the analyzed triticale cultivar had exceeded the productivity of the concrete wheat cultivar and had performed better under water-limited conditions [32]. The plants were grown for 17 days under controlled conditions (16/8 h photoperiod with day/night temperatures of 22 °C/17 °C and 60% relative air humidity) on leached meadow cinnamon soil (540 g, pH 6.2) in pots with a capacity of 500 cm^3^, each containing 20 individuals. The seedlings (with a second fully expanded and third developing leaf) were sprayed once with the selective herbicide (Serrate^®^, Syngenta, Sofia, Bulgaria), applying the standard dose recommended by the manufacturer (25 g per 1000 m^2^). The herbicide formulation contains the active compounds clodinafop-propargyl (20%) and pyroxsulam (7.5%), as well as cloquintocet-mexyl (7.5%), acting as a safener. Seventy-two hours later, some of the controls and part of the herbicide-treated plants were subjected to drought [38] or waterlogging [47] for seven consecutive days, as this period was sufficient to provoke severe but recoverable stress. The recovery of the stressed plants was performed for 96 h by resuming the normal water supply. Samples from the different treatment groups (D—drought, HD—herbicide and drought, W—waterlogging, HW—herbicide and waterlogging) and the relevant controls (C—controls; H—herbicide-treated and grown under normal conditions) were collected at 0 h (3 days after the application of the herbicide), 96 h, and 168 h of drought or waterlogging, and at the end of the recovery period. The scheme of the experimental setup is represented in Figure S2.

The fresh weight and the length of the aboveground plant parts were determined immediately after harvesting. The dry weight was measured after keeping the samples for several days in an oven set at 80 °C until reaching a constant weight. In parallel, bulked leaf samples (100 mg derived from the first and second fully expanded true leaves) were frozen in liquid nitrogen and stored at −80 °C until qRT-PCR analyses.

### 4.2. Histochemical Detection of O_2_^●−^and H_2_O_2_

The histochemical detection of O_2_^●−^and H_2_O_2_ was performed with nitroblue tetrazolium (NBT) and 3,3′ -diaminobenzidine (DAB) staining, respectively, according to the procedure described in [50]. The second leaf (L2), which was the last fully expanded one at the herbicide treatment stage, was sampled for NBT and DAB staining at each experimental point (see the scheme in Figure S2). At the recovery stage, histochemical detection of O_2_^●−^ and H_2_O_2_ was also performed on the third leaf (L3), which was developing and reached full expansion at the end of the experimental period. The staining procedure started with cutting the leaves from each treatment group into three equal parts. The middle leaf segments were used in the staining procedure. They were submerged in freshly prepared 0.05% (*w*/*v*) NBT in 50 mM Na-PO_4_ buffer (pH 7.4) to detect O_2_^●−^, or in 1 mg/mL DAB dissolved in 10 mM Na_2_HPO_4_ for H_2_O_2_ staining. The samples were incubated in a vacuum container in darkness for 1 h at room temperature. The leaf segments were cleared in a solution of ethanol:acetic acid:glycerol (3:1:1 ratio) for 24 h at room temperature. Then, they were transferred to 100% lactic acid and kept in darkness at 4 °C until analyses (usually within 48 h following the staining and distaining procedures).

Leaf segment white field images were obtained with a binocular microscope (BMS74955, Breukhoven, Rotterdam, Netherlands) fitted with a digital camera (5MP CMOS USB). The images were analyzed with the “Colour Deconvolution 2” plugin for ImageJ [51] based on the protocol described in [52]. The quantification of the staining intensity signals (Figure 2 and Figure 3) is expressed as the optical density (OD), calculated according to the formula OD = log(max intensity/mean intensity), where the maximum intensity of 8-bit images is equal to 255 (in an 8-bit digital image, 0 = black and 255 = white).

### 4.3. RT-PCR Analysis

Total RNA was extracted using the GeneJET Plant RNA Purification Kit (Thermo Scientific, Waltham, MA, USA). The concentration of the RNA samples was measured with a micro-UV-VIS spectrophotometer, Nano Drop 2000 (Thermo Scientific, Basel, Switzerland). Reverse transcription was performed with 1 μg total RNA using the Scriptase RT—cDNA Synthesis Kit (GENAXXON Bioscience, Ulm, Germany) according to the manufacturer’s instructions. The gene transcript abundance was evaluated by quantitative real-time RT-PCR (qRT-PCR) using 2X GreenMasterMix No ROX^TM^ (GENAXXON Bioscience, Ulm, Germany) with the ‘PikoReal’ Real-Time PCR System (Thermo Scientific, Basel, Switzerland). The PCR program settings were 95 °C for 15 min and 45 cycles of 95 °C for 15 s followed by 55 °C–60 °C for 30 s, and melting curve analysis with a temperature range of 60 °C–95 °C in 0.2 °C increments for 60 s.

The sequences of the analyzed *Triticum aestivum* genes are publicly available in the NCBI database (National Center of Biotechnology Information). The genes coding for superoxide dismutase enzymes included *SOD1.2* (a nuclear gene encoding a chloroplast Cu/Zn SOD, U69632.1); *FeSOD* (a chloroplastic gene for iron-superoxide dismutase, LOC101290631); *SOD* (a nuclear gene encoding mitochondrial manganese superoxide dismutase LOC542833); *CAT3* (catalase isozyme 2, LOC100682478) and *CATA* (catalase, LOC543316); *GR* (glutathione reductase, GR305072); *POD1* (ascorbate peroxidase, FJ890988.1); *POX2* (class III peroxidase, LOC543313), *P5CS1* (delta-1-pyrroline-5-carboxylate synthase 1, LOC606368); *P5CR* (pyrroline-5-carboxylate reductase, LOC606347). The relative expression of the target genes was calculated using the ΔΔCq method [53],with *alpha tubulin* (U76558.1), *18S ribosomal RNA* (LOC123171822), and *elongation factor-1 alpha* (LOC123123039) as reference genes. The primer sequences are presented in Table 1.

### 4.4. Statistical Analyses

The presented results (from three independent experiments) are based on a completely randomized design. At least 15 individuals per treatment group were used for the measurements of the growth parameters (FW, DW, and stem length). The qRT-PCR data were derived from three biological replicates per experimental group. Statistical analyses were performed in Excel, including one-way ANOVA, *t*-test (*n* = 3), and post hoc Tukey HSD analyses (*n* ≥ 15) (Tables S1 and S2). The error bars in the graphs reflect the standard error (SE) or standard deviation (SD).

## 5. Conclusions

Commonly applied weed-managing products could be beneficial for improved plant performance under certain unfavorable conditions, but their use may also have negative consequences that depend on the crop and stress type. We identified some elements of the antioxidant defense system that might be related to the divergent capacity of winter wheat and the hybrid crop triticale to tolerate adverse water supply after the application of a conventional dose of a selective herbicide. Waterlogged triticale showed considerably higher expression of two catalase and two peroxidase coding genes compared to wheat, which correlates with its better performance under excessive water regime. The hybrid crop had more stable transcript levels of the glutathione reductase gene, which could also contribute to its better capacity to detoxify ROS through the glutathione defense system. We conclude that the administration of selective herbicides in winter wheat fields that are prone to waterlogging should be considered after carefully evaluating the risks and benefits, as the herbicide treatment could enhance the negative consequences of excessive water stress. In contrast, although the hybrid crop triticale can tolerate the combination of herbicide application with subsequent waterlogging events, its drought resilience seemed to be negatively influenced by the treatment with the tested agrochemical product, especially after prolonged water deprivation.

## Figures and Tables

**Table 1:** Primer pairs used in the qRT-PCR analyses.

Gene Name	Locus	Forward Primer (5′-3′)	Reverse Primer (5′-3′)
*Cu/Zn SOD*	U69632.1	ttaacccaaacggcctgacacat	caacaaacgctctcccaacaactg
*Mn SOD*	LOC542833	cgccacctacgtcgcccactac	acatgaccgccgccgttgaa
*Fe SOD*	LOC101290631	gtctggttgggtttggcttgtctt	ttcgcctgtcatccttgtaatcca
*CAT3*	LOC100682478	caccctcgtcggcggcaagaac	cacgggctggagggggacgag
*CATA*	LOC543316	gggagccagtgcaaagggattc	cacggtcatgcacaacggtagaga
*GR*	GR305072	gttgaagtcacccagccaga	tccgccaccaagaatcacag
*POD1*	FJ890988.1	caaggctctgaccacctcag	catcttcccagggtgtgacc
*POX2*	LOC543313	gcggtgacaccaacatcaac	gtccaggttctccaggttgg
*P5CS*	LOC606368	ctctacagcggtccaccaag	caggtacaccacccgttgaa
*P5CR*	LOC606347	taaatgccgttgttgctgcc	agcaaaactaacaatggctaccag
*α-TUB*	U76558.1	ttctcccgcatcgaccacaagttt	tcatcgccctcatcaccgtcc
*18S RNA*	LOC123171822	tacctggttgatcctgccagt	caatgatccttccgcaggttcac
*EF-1 α*	LOC123123039	cagatcggcaacggctac	gagaaggtctccaccaccat

## Data Availability

The data presented in this study are available on request from the corresponding author.

## Supplementary Materials

The supporting information can be downloaded at: https://www.mdpi.com/article/10.3390/ijms241512503/s1.
