# Supplementary material for: Divergent Cross-Adaptation of Herbicide-Treated Wheat and Triticale Affected by Drought or Waterlogging
Source: Int J Mol Sci. 2023 Aug 6;24(15):12503. doi: 10.3390/ijms241512503 (PMC10419764; doi:10.3390/ijms241512503)
Supplement: Supplementary file 1 [file ijms-24-12503-s001.zip › ijms-2502417-supplementary.pdf]

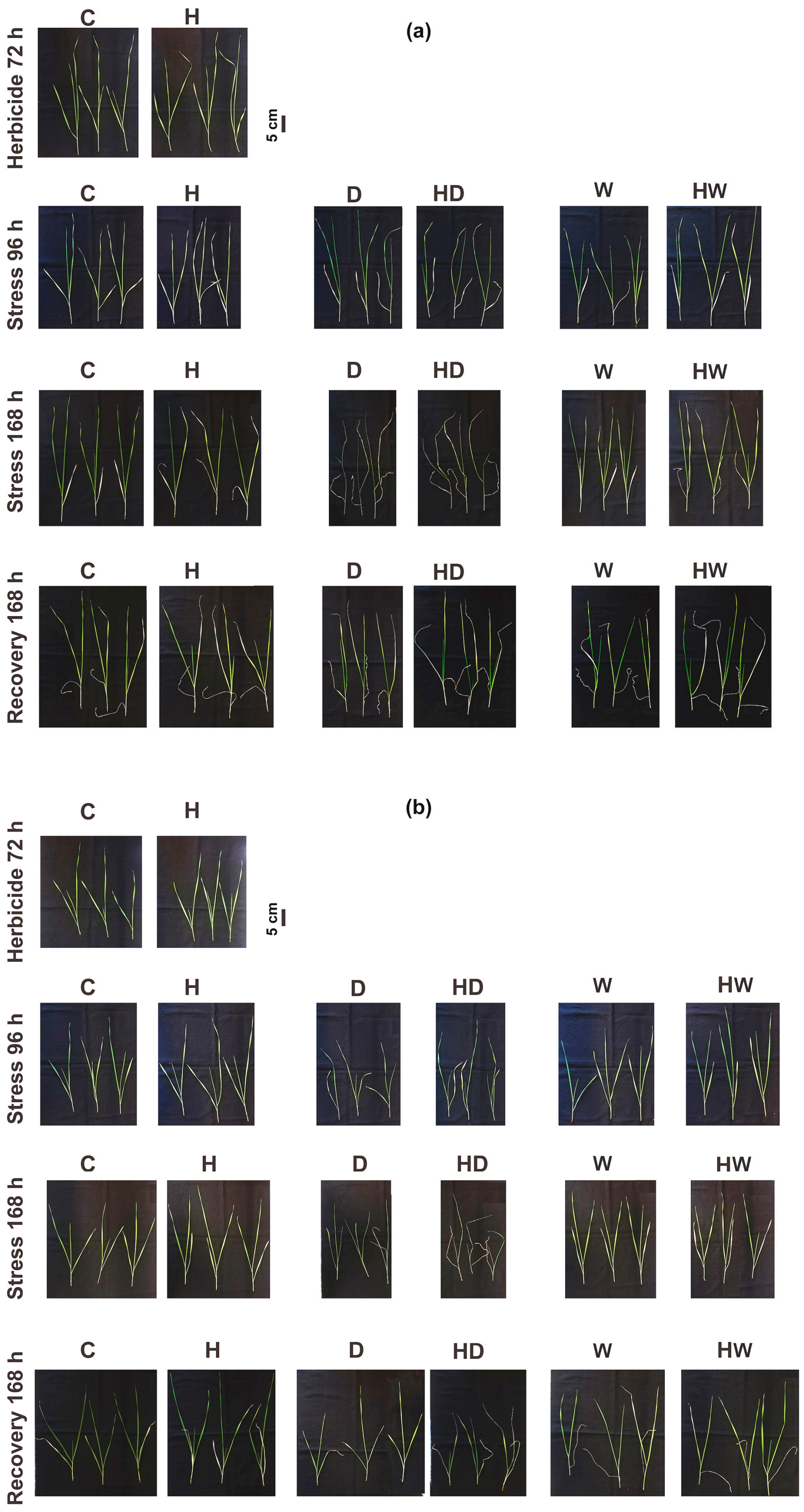

Figure S1. Phenotype of wheat (a) and triticale (b) control plants (C) and plants subjected to standard dose of selective herbicide Serrate® (H), drought (D) or waterlogging (W) and their combination (HD and HW respectively).

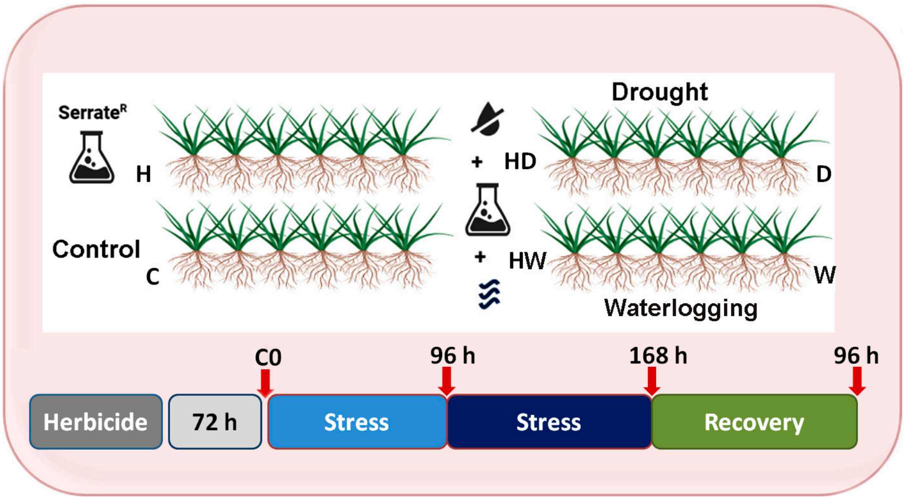

Figure S2. Experimental model. The red arrows indicate the sampling points included in the study. (C- controls; H-herbicide treated, a single standard dose has been applied at the beginning of the experiment; D-drought; W- waterlogged; HD – herbicide treated and subjected to drought; HW – herbicide treated and subjected to waterlogging).

Table S1. Tukey HSD (Honestly Significant Difference) analyses of wheat biometric measurements, at 96 h of stress, 168 h of stress and after 96 h recovery period. C – controls; H- herbicide treated; D – drought; HD – herbicide treated and subjected to drought; W – waterlogging; HW – herbicide treated and subjected to waterlogging. (“insig.” – insignificant difference, red box; green boxes represent statistically significant differences): **(A) Fresh weight; (B) Dry weight**; **(C) Shoot length.**

(A) Fresh weight – wheat

Treatment pair	96 h Stress Tukey HSD			168 h Stress Tukey HSD			96 h Recovery Tukey HSD		
	Q statistic	p-value	inferfence	Q statistic	p-value	inferfence	Q statistic	p-value	inferfence
C vs H	1.422	0.9	insig.	0.716	0.9	insig.	2.132	0.641	insig.
C vs D	7.314	0.001	** p<0.01	15.23	0.001	** p<0.01	15.14	0.001	** p<0.01
C vs HD	7.366	0.001	** p<0.01	15.38	0.001	** p<0.01	14.39	0.001	** p<0.01
C vs W	0.761	0.9	insig.	7.767	0.001	** p<0.01	12.02	0.001	** p<0.01
C vs HW	0.028	0.9	insig.	8.48	0.001	** p<0.01	15.42	0.001	** p<0.01
H vs D	8.304	0.001	** p<0.01	15.92	0.001	** p<0.01	13.21	0.001	** p<0.01
H vs HD	8.367	0.001	** p<0.01	16.09	0.001	** p<0.01	12.31	0.001	** p<0.01
H vs W	0.704	0.9	insig.	8.483	0.001	** p<0.01	9.888	0.001	** p<0.01
H vs HW	1.395	0.9	insig.	9.196	0.001	** p<0.01	13.29	0.001	** p<0.01
D vs HD	0.144	0.9	insig.	0.261	0.9	insig.	1.693	0.816	insig.
D vs W	8.056	0.001	** p<0.01	7.667	0.001	** p<0.01	4.236	0.046	* p<0.05
D vs HW	7.342	0.001	** p<0.01	6.974	0.001	** p<0.01	1.152	0.9	insig.
HD vs W	8.128	0.001	** p<0.01	7.609	0.001	** p<0.01	2.686	0.416	insig.
HD vs HW	7.395	0.001	** p<0.01	6.897	0.001	** p<0.01	0.622	0.9	insig.
W vs HW	0.733	0.9	insig.	0.712	0.9	insig.	3.399	0.175	insig.

(B) Dry weight – wheat

Treatment pair	96 h Stress Tukey HSD			168 h Stress Tukey HSD			96 h Recovery Tukey HSD		
	Q statistic	p-value	inferfence	Q statistic	p-value	inferfence	Q statistic	p-value	inferfence
C vs H	0.128	0.9	insig.	2.995	0.322	insig.	5.786	0.009	** p<0.01
C vs D	2.172	0.631	insig.	3.158	0.271	insig.	7.931	0.001	** p<0.01
C vs HD	1.342	0.9	insig.	3.594	0.164	insig.	6.423	0.004	** p<0.01
C vs W	3.77	0.132	insig.	2.178	0.629	insig.	1.365	0.9	insig.
C vs HW	4.281	0.067	insig.	2.451	0.526	insig.	3.185	0.268	insig.
H vs D	2.3	0.583	insig.	6.154	0.004	** p<0.01	13.72	0.001	** p<0.01
H vs HD	1.47	0.897	insig.	6.589	0.002	** p<0.01	11.15	0.001	** p<0.01
H vs W	3.642	0.155	insig.	0.817	0.9	insig.	7.151	0.001	** p<0.01
H vs HW	4.153	0.08	insig.	0.545	0.9	insig.	8.971	0.001	** p<0.01
D vs HD	0.831	0.9	insig.	0.436	0.9	insig.	0.053	0.9	insig.
D vs W	5.942	0.006	** p<0.01	5.337	0.015	* p<0.05	6.566	0.003	** p<0.01
D vs HW	6.453	0.003	** p<0.01	5.609	0.01	** p<0.01	4.746	0.039	* p<0.05
HD vs W	5.112	0.021	* p<0.05	5.772	0.008	** p<0.01	5.308	0.018	* p<0.05
HD vs HW	5.623	0.01	** p<0.01	6.045	0.005	** p<0.01	3.822	0.129	insig.
W vs HW	0.511	0.9	insig.	0.272	0.9	insig.	1.82	0.765	insig.

(C) Shoot length – wheat

Treatments pair	96 h Stress Tukey HSD			168 h Stress Tukey HSD			96 h Recovery Tukey HSD		
	Q statistic	p-value	inferfence	Q statistic	p-value	inferfence	Q statistic	p-value	inferfence
**C vs H**	0.407	0.9	insig.	0.373	0.9	insig.	0.149	0.9	insig.
**C vs D**	4.878	0.011	* p<0.05	5.895	0.001	** p<0.01	5.216	0.005	** p<0.01
**C vs HD**	4.159	0.047	* p<0.05	5.602	0.002	** p<0.01	4.785	0.014	* p<0.05
**C vs W**	1.506	0.891	insig.	2.604	0.448	insig.	4.694	0.017	* p<0.05
**C vs HW**	2.61	0.445	insig.	3.3	0.193	insig.	5.525	0.003	** p<0.01
**H vs D**	4.11	0.052	insig.	5.32	0.004	** p<0.01	5.234	0.005	** p<0.01
**H vs HD**	3.459	0.153	insig.	5.052	0.008	** p<0.01	4.775	0.014	* p<0.05
**H vs W**	0.997	0.9	insig.	2.136	0.639	insig.	4.705	0.016	* p<0.05
**H vs HW**	2.033	0.68	insig.	2.798	0.365	insig.	5.568	0.002	** p<0.01
**D vs HD**	0.64	0.9	insig.	0.176	0.9	insig.	0.081	0.9	insig.
**D vs W**	3.293	0.195	insig.	3.336	0.183	insig.	0.78	0.9	insig.
**D vs HW**	2.103	0.652	insig.	2.744	0.387	insig.	0.041	0.9	insig.
**HD vs W**	2.611	0.444	insig.	3.093	0.255	insig.	0.783	0.9	insig.
**HD vs HW**	1.453	0.9	insig.	2.509	0.488	insig.	0.122	0.9	insig.
**W vs HW**	1.113	0.9	insig.	0.655	0.9	insig.	0.793	0.9	insig.

Table S2. Tukey HSD (Honestly Significant Difference) analyses of triticale biometric measurements, at 96 h of stress, 168 h of stress and after 96 h recovery period. C – controls; H- herbicide treated; D – drought; HD – herbicide treated and subjected to drought; W – waterlogging; HW – herbicide treated and subjected to waterlogging. ("insig." – insignificant difference, red boxes; green boxes represent statistically significant differences): **(A) Fresh weight; (B) Dry weight**; **(C) Shoot length.**

(A) Fresh weigh - triticale

Treatment pair	96 h Stress Tukey HSD			168 h Stress Tukey HSD			96 h Recovery Tukey HSD		
	Q statistic	p-value	inferfence	Q statistic	p-value	inferfence	Q statistic	p-value	inferfence
**C vs H**	1.984	0.7	insig.	0.319	0.9	insig.	1.758	0.79	insig.
**C vs D**	12.03	0.001	** p<0.01	19.86	0.001	** p<0.01	17.05	0.001	** p<0.01
**C vs HD**	11.24	0.001	** p<0.01	20.44	0.001	** p<0.01	17	0.001	** p<0.01
**C vs W**	2.14	0.636	insig.	0.291	0.9	insig.	3.962	0.065	insig.
**C vs HW**	0.65	0.9	insig.	0.677	0.9	insig.	3.077	0.257	insig.
**H vs D**	11.6	0.001	** p<0.01	20.19	0.001	** p<0.01	18.59	0.001	** p<0.01
**H vs HD**	10.69	0.001	** p<0.01	20.77	0.001	** p<0.01	18.54	0.001	** p<0.01
**H vs W**	0.306	0.9	insig.	0.018	0.9	insig.	5.626	0.002	** p<0.01
**H vs HW**	1.384	0.9	insig.	0.962	0.9	insig.	4.795	0.012	* p<0.05
**D vs HD**	0.909	0.9	insig.	0.122	0.9	insig.	0.046	0.9	insig.
**D vs W**	10.43	0.001	** p<0.01	19.55	0.001	** p<0.01	12.63	0.001	** p<0.01
**D vs HW**	12.12	0.001	** p<0.01	17.03	0.001	** p<0.01	13.97	0.001	** p<0.01
**HD vs W**	9.592	0.001	** p<0.01	20.07	0.001	** p<0.01	12.59	0.001	** p<0.01
**HD vs HW**	11.28	0.001	** p<0.01	17.4	0.001	** p<0.01	13.93	0.001	** p<0.01
**W vs HW**	1.581	0.862	insig.	0.923	0.9	insig.	0.967	0.9	insig.

(B) Dry weight - triticale

Treatment pair	96 h Stress Tukey HSD			168 h Stress Tukey HSD			96 h Recovery Tukey HSD		
	Q statistic	p-value	inferfence	Q statistic	p-value	inferfence	Q statistic	p-value	inferfence
C vs H	1.304	0.9	insig.t	0.11	0.9	insig.t	4.185	0.079	insig.t
C vs D	0.401	0.9	insig.t	2.525	0.499	insig.t	8.477	0.001	** p<0.01
C vs HD	0.602	0.9	insig.t	2.58	0.478	insig.t	9.604	0.001	** p<0.01
C vs W	3.209	0.256	insig.t	1.067	0.9	insig.t	0.662	0.9	insig.t
C vs HW	4.312	0.064	insig.t	0.878	0.9	insig.t	0.107	0.9	insig.t
H vs D	1.705	0.808	insig.t	2.415	0.541	insig.t	12.66	0.001	** p<0.01
H vs HD	1.905	0.732	insig.t	2.47	0.52	insig.t	13.79	0.001	** p<0.01
H vs W	1.905	0.732	insig.t	1.169	0.9	insig.t	4.537	0.049	* p<0.05
H vs HW	3.008	0.317	insig.t	0.988	0.9	insig.t	4.078	0.091	insig.t
D vs HD	0.201	0.9	insig.t	0.055	0.9	insig.t	1.127	0.9	insig.t
D vs W	3.61	0.161	insig.t	3.404	0.208	insig.t	7.186	0.001	** p<0.01
D vs HW	4.713	0.037	* p<0.05	3.403	0.209	insig.t	8.584	0.001	** p<0.01
HD vs W	3.811	0.125	insig.t	3.455	0.196	insig.t	8.229	0.001	** p<0.01
HD vs HW	4.914	0.028	* p<0.05	3.458	0.196	insig.t	9.711	0.001	** p<0.01
W vs HW	1.103	0.9	insig.t	0.254	0.9	insig.t	0.762	0.9	insig.t

(C) Shoot length - triticale

Treatment pair	96 h Stress			168 h Stress			96 h Recovery		
	Tukey HSD			Tukey HSD			Tukey HSD		
	Q statistic	p-value	inferfence	Q statistic	p-value	inferfence	Q statistic	p-value	inferfence
**C vs H**	1.042	0.9	insig.t	0.978	0.9	insig.t	0.083	0.9	insig.t
**C vs D**	2.796	0.363	insig.t	3.288	0.194	insig.t	4.045	0.056	insig.t
**C vs HD**	3.461	0.149	insig.t	3.73	0.098	insig.t	5.071	0.007	** p<0.01
**C vs W**	0.183	0.9	insig.t	1.107	0.9	insig.t	1.952	0.712	insig.t
**C vs HW**	1.213	0.9	insig.t	1.189	0.9	insig.t	1.082	0.9	insig.t
**H vs D**	3.949	0.066	insig.t	2.182	0.62	insig.t	4.013	0.059	insig.t
**H vs HD**	4.649	0.017	* p<0.05	2.538	0.475	insig.t	5.051	0.007	** p<0.01
**H vs W**	0.898	0.9	insig.t	0.028	0.9	insig.t	1.896	0.735	insig.t
**H vs HW**	0.164	0.9	insig.t	0.142	0.9	insig.t	1.014	0.9	insig.t
**D vs HD**	0.65	0.9	insig.t	0.248	0.9	insig.t	1.204	0.9	insig.t
**D vs W**	3.102	0.249	insig.t	2.385	0.538	insig.t	1.888	0.738	insig.t
**D vs HW**	4.163	0.045	* p<0.05	2.179	0.621	insig.t	2.893	0.324	insig.t
**HD vs W**	3.801	0.086	insig.t	2.813	0.356	insig.t	2.959	0.3	insig.t
**HD vs HW**	4.876	0.01	* p<0.05	2.571	0.461	insig.t	3.953	0.066	insig.t
**W vs HW**	1.075	0.9	insig.t	0.128	0.9	insig.t	0.892	0.9	insig.t
